# Fully Automated Molecular Diagnostic System “Simprova” for Simultaneous Testing of Multiple Items

**DOI:** 10.1038/s41598-020-62109-5

**Published:** 2020-03-25

**Authors:** Toshihiro Yonekawa, Hidetoshi Watanabe, Norimitsu Hosaka, Shohei Semba, Atsushi Shoji, Masaki Sato, Masato Hamasaki, Shota Yuki, Shiori Sano, Yuji Segawa, Tsugunori Notomi

**Affiliations:** Biochemical Research Laboratory II, Research & Development Division, Eiken Chemical Co., Ltd. 143 Nogi Nogimachi, Shimotsuga-gun, Tochigi 329-0114 Japan

**Keywords:** Biochemistry, Biological techniques, Cancer, Microbiology, Molecular biology

## Abstract

Nucleic acid amplification-based diagnostics is known as one of the molecular diagnostic systems that allows higher sensitive detection of pathogens than test methods such as immunoassay. However, it has not been widely used because it is complicated to use and takes a long time to generate results. On the other hand, development of fully automated molecular diagnostic systems has been growing around the world as demand for such systems from physicians and laboratory technicians has increased. To meet this demand, we have developed the “Simprova” fully automated molecular diagnostic system, which takes advantage of LAMP (Loop-mediated Isothermal Amplification), a method Eiken Chemical Co., Ltd. invented. Simprova comprises a master unit that controls the entire system and a test unit that extracts and purifies nucleic acid from samples (pretreatment), and uses the LAMP method to detect and amplify nucleic acid. Users can obtain test results automatically by simply installing a pretreatment cartridge, a multi-well testing chip and the sample in the test unit. The multi-well testing chip has 25 reaction wells connected by channels and enables simultaneous testing of multiple targets with one sample. Turnaround time for one test is approximately 30 minutes. Since a conventional extraction and purification method using magnetic-bead separation is used for the pretreatment, nucleic acid can be extracted from serum, plasma, whole blood, urine, and sputum, for example. In addition, the system can perform random-access testing by connecting four test units to the master unit to realize near-the-patient testing. Simprova is therefore a robust and useful system for a wide variety of applications.

## Introduction

Nucleic acid-based diagnosis can be used to predict a disease possibility and determine a treatment effect through analysis of human genetic information and detection of abnormality of the genes. It also identifies genes of pathogens such as virus and bacteria to determine the cause of a disease and can be used for determination of medical treatment and its effect. Major methods of gene analysis include a detection method to amplify a small amount of nucleic acid in a test tube and use a probe having high specificity (nucleic acid amplification test, NAAT), a micro-array method (DNA chip method) to bond an amplified nucleic acid to various detection target nucleic acids densely immobilized on a plastic or glass substrate, and a sequencing to clarify the order of base sequences constituting nucleic acids. Each method is used properly depending on the purpose of analysis. In particular, NAAT is widely used for clinical diagnosis of infectious diseases as it realizes rapid and highly sensitive detection.

A variety of nucleic acid amplification techniques has been developed since a typical method, Polymerase Chain Reaction (PCR) method^1–3^, was developed. On the other hand, there are isothermal amplification methods using the strand replacement activity of DNA polymerase, such as loop-mediated isothermal amplification (LAMP) method^4^ and strand displacement amplification (SDA) method^5^. Furthermore, there are amplification methods of using T7 RNA polymerase to obtain final amplified products of RNA, such as nucleic acid sequence-based amplification (NASBA) method^6^, transcription-mediated amplification (TMA) method^7^, and transcription-reverse transcription concerted (TRC) method^8^. In particular, the LAMP method is commonly used at many clinical sites and research institutes because of its simplicity, high amplification efficiency and high specificity.

Meanwhile, Immunochromatographic Assay (ICA) is widely employed as an easy-to-use Rapid Diagnosis Test (RDT) for diagnosing infectious disease in medical practice. However, it has problems such as false-positives and false-negatives due to differences among individuals when conducting visual checks, and false-negatives due to testing in the early stage of infection when the number of pathogens is small^9^. Therefore, nucleic acid-based diagnosis is effective to resolve these problems.

Among recent technologies for nucleic acid-based diagnosis, various products of fully automated testing systems which realize simultaneous testing of multiple items are getting popular in the US and Europe^10^. The simultaneous testing of multiple items have many advantages. Since it can identify a pathogen for infectious diseases in a single test, reduction of patient’s burden of sample collecting, testing time and medical expenses can be expected. It is also useful for detection of multiple genes or multiple mutations to choose a method of cancer treatment. Moreover, simultaneous measurement of control genes ensures accuracy of test process and sample collection in many systems^11,12^.

We have developed technologies focusing on simultaneous testing of multiple items to expand applications of the nucleic acid-based diagnosis. Employing a specific fluorescently labeled probe, we developed an amplification and detection chip enabling simultaneous detection of multiple items of genes including control genes and a compact testing system with full automated functions of extraction and purification of nucleic acid from the sample and of amplification by LAMP method and detection.

At present, there is a fully automated system in the market made by Sysmex Corporation, called RD-100i^13^, for detecting nucleic acid using the LAMP method. That system applies a simplified nucleic acid extraction method and turbidity measurement method to detect coproducts raised from nucleic acid amplification. Several miniaturized LAMP detection systems and microfluidics applying LAMP method are also undergoing R&D^14^, but they have not been commercialized yet.

In this paper, we report about our newly developed fully-automated molecular diagnostic system ‘Simprova’, which realizes simultaneous detection of multiple items using LAMP method with high-speed nucleic acid extraction and purification, and its performance.

## Results

### Basic performance of pretreatment

**Figure 1:**
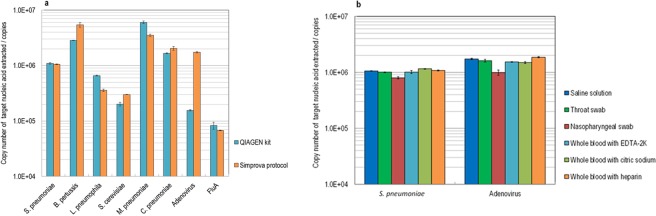
Extraction performance using various pathogens. (**a**) The pathogens used for nucleic acid extraction test were spiked into saline in the following amounts: *S. pneumoniae*, *B. pertussis*, and *L. pneumoniae* of 2 μL at McF#1 concentration, *M. pneumoniae*, *C. pneumoniae*, Adenovirus, and FluA of 2 μL of exponential growth phase culture medium, and *S. cerevisiae* of 3 μL of culture medium at OD 600 nm = 6.0. Nucleic acid extraction was performed twice by the QIAGEN method and three times by the Simprova method. (**b**) *S. pneumoniae* and adenovirus were spiked into various specimens. The amount of pathogen spiked is the same as in (a) Each test was performed n = 3 with saline, n = 5 with throat swabs, n = 5 with nasopharyngeal swabs, n = 3 with whole blood containing EDTA-2K, n = 3 with whole blood containing sodium citrate, and n = 3 with whole blood containing heparin. The error bar in each graph represents the standard deviation from repeated tests.

Various pathogens and yeast were spiked to the saline and nucleic acid was extracted and purified by using the newly developed protocol. Then the performance of extracted nucleic acid was evaluated with the quantitative PCR. The process time of the QIAGEN-made kit was 40 minutes or longer, while the new extraction method, the “Simprova protocol,” took only 15 minutes to achieve similar performance of the nucleic acid extraction (Fig. 1a). There was no statistically significant difference between the number of copies of *S. pneumoniae*, *B. pertussis*, *C. pneumoniae* and FluA extracted using the QUIAGEN extraction kit and by the Simprova protocol according to a t-test at the p = 0.05 level. However, the number of copies of *C. pneumoniae* and Adenovirus extracted using the Simprova protocol appeared to be more than by the QUIAGEN extraction kit. The p level by the t-test in the number of copies of *L. pneumophila* and *M. pneumoniae* extracted between the Simprova protocol and QUIAGEN-made kit was less than 0.05, but the difference was less than twofold.

For performance comparison of the extraction from various samples, typical pathogens, pneumococcus and adenovirus, were spiked to each sample and the performance of the nucleic acid extraction was evaluated by the quantitative PCR. The result indicated that the extraction performance was almost in a similar level irrespective of the sample type without receiving influence from sample components (Fig. 1b). It was only in the case of the nasopharyngeal swab that the number of copies extracted using the Simprova protocol was relatively smaller than other cases for both pathogens, but the difference was not more than twofold.

### Basic performance of LAMP amplification test using multi-well testing chip

A respiratory infection disease panel containing reagents to detect *Bordetella pertussis* (BP), *Mycoplasma pneumoniae* (MP), *Chlamydophila pneumoniae* (CP) and *Legionella pneumophila* (LP) was developed as multi-well testing chip. First, a plasmid DNA having target sequences of BP, MP, CP and LP was serially diluted and a test for limit of detection (LoD) was performed. As a result, LoD of any target was 4 copies or less (Table 1). Next, the bacterial genomes purified from ATCC strain and clinical isolate were used to perform tests for specificity and cross-reactivity. As a result of the specificity test using 10 strains of BP, 5 strains of MP, 6 strains of CP and 23 strains of LP, only the target bacteria were detected with no cross-reaction. No cross-reaction with other 46 pathogenic bacterial genomes was found (data not shown).

**Table 1:** Limit of detection (LoD) test result.

	cps/well	BP					MP					CP					LP				
		1st	2nd	3rd	4th	5th	1st	2nd	3rd	4th	5th	1st	2nd	3rd	4th	5th	1st	2nd	3rd	4th	5th
Dilution 1	15.00	5	5	5	5	5	5	5	5	5	5	5	5	5	5	5	5	5	5	5	5
	7.50	4	5	5	4	4	5	5	5	5	5	3	4	4	5	3	5	5	5	5	5
	3.75	5	4	4	3	3	4	4	4	3	5	3	4	4	4	5	5	4	4	5	4
	1.88	2	3	3	2	3	2	3	4	3	3	1	3	2	2	3	2	3	3	1	1
	0.94	2	3	1	2	3	2	2	3	2	1	1	2	**0**	2	**0**	1	3	1	2	1
Dilution 2	12.50	5	5	5	5	5	5	5	5	5	5	5	5	5	5	5	5	5	5	5	5
	6.25	4	5	5	5	5	4	5	5	5	5	4	2	3	4	3	3	5	5	5	5
	3.13	5	5	5	5	3	3	4	4	3	3	2	5	**0**	3	3	4	3	4	5	3
	1.56	2	3	3	2	4	2	4	3	2	3	2	**0**	2	1	1	4	3	2	2	2
	0.78	2	1	2	3	3	2	1	1	1	2	**0**	1	**0**	**0**	3	1	2	2	2	2
Dilution 3	10.00	5	5	5	5	5	4	5	5	5	5	5	5	4	5	4	5	5	5	5	5
	5.00	5	5	5	5	5	4	5	4	4	5	3	4	4	4	3	3	3	5	5	5
	2.50	4	2	3	5	4	4	4	4	2	4	3	3	2	3	3	3	1	2	1	4
	1.25	2	2	2	2	**0**	2	5	3	2	3	1	1	1	2	1	3	1	2	1	4
	0.63	3	1	3	1	2	2	2	2	2	1	1	1	1	2	**0**	1	2	2	1	2
	0	0	0	0	0	0	0	0	0	0	0	0	0	0	0	0	0	0	0	0	0
	**LoD**	**1.56 cps/well**					**< 0.63 cps/well**					**3.75 cps/well**					**< 0.63 cps/well**				

The solutions, including the target template of each item, were diluted in stages from concentrations of 25 copies/well, 15 copies/well and 10 copies/well, respectively, and used for LoD tests. LoD is defined as the number of copies present after concentrating the copies in one more step in the case that all five wells were negative when the concentration test was performed five times.

The solutions, including the target template of each item, were diluted in stages from concentrations of 25 copies/well, 15 copies/well and 10 copies/well, respectively, and used for LoD tests. LoD is defined as the number of copies present after concentrating the copies in one more step in the case that all five wells were negative when the concentration test was performed five times.

### Basic performance of fully automated nucleic acid-based diagnosis system

**Figure 2:**
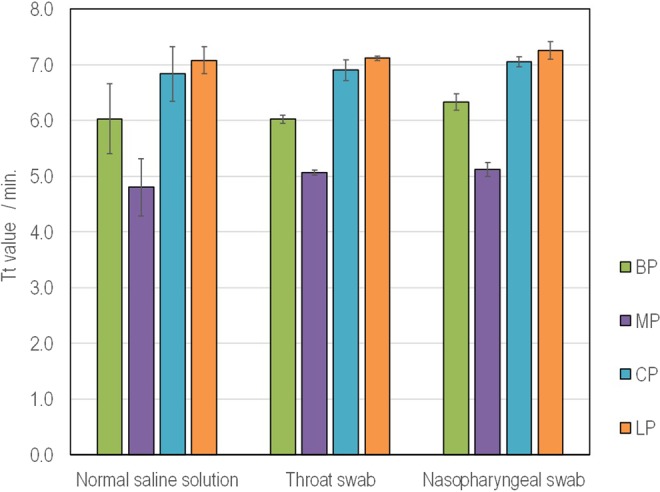
Simultaneous detection results of multiple items by Simprova system. The amount of pathogen added to each sample solution is the same as in Fig. 1a. The Tt value is an average of 5 wells and the number of tests is n = 3. The error bar in each graph represents the standard deviation of repeated tests.

**Figure 3:**
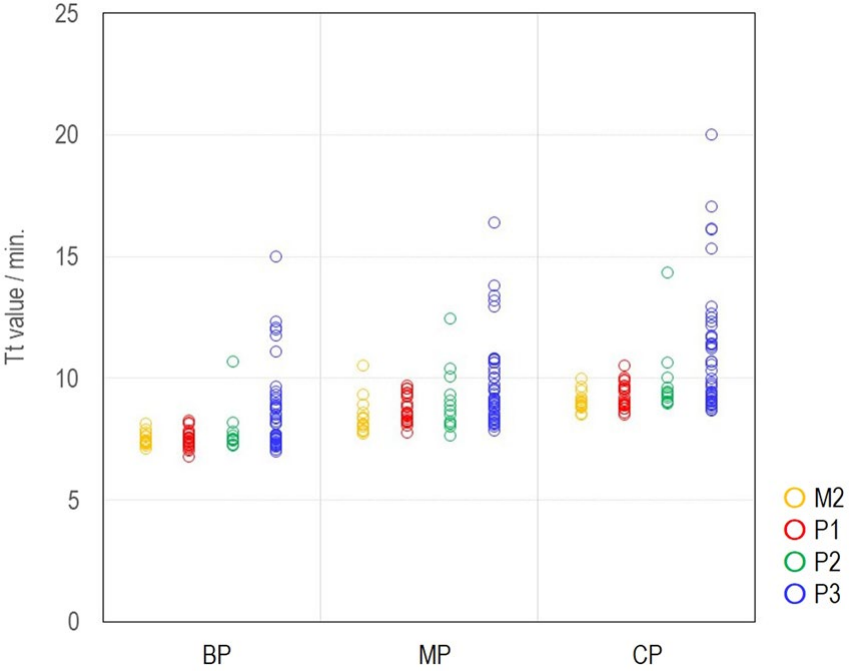
Detection results from 101 kinds of sputum spiked. Refer to Fig. 6 for 101 sputum classification. The amount of pathogen contained in each sample was 1250 copies of MP, 1250 copies of CP and 1875 copies of LP, and the average value of detection time (Tt) of 5 wells was plotted for each item.

To examine the performance of the fully-automated processes from pretreatment to amplification and detection, bacterial cells of BP, MP, CP and LP were spiked to expected samples of saline, throat swab and nasopharyngeal swab. There was no statistically significant difference found among the samples by the t-test at the P = 0.05 level, and all bacteria could be automatically detected in all wells (Fig. 2). Moreover, pathogens (CP, LP and MP) were spiked to 101 kinds of sputum in various properties to evaluate the performance of the direct sputum processing. The more purulent sputum samples had a tendency toward the longer detection time. However, the nucleic acid solution extracted by the new extraction technique did not strongly inhibit the reaction and detection finished in 20 minutes. Therefore, the automatic detection including the sputum processing finished in 35 minutes (Fig. 3).

## Discussion

We have developed a new fully-automated compact nucleic-acid-based diagnosis system “Simprova” taking advantage of LAMP. We evaluated this system by taking basic analytical data using a newly developed pretreatment technique and a multi-well testing chip to detect various kinds of pathogens. The performance of the new pretreatment technique is in the similar level as the QIAGEN extraction kit which is widely used at many clinical sites and the technique can handle various samples such as swab, serum, plasma, whole blood and urine, etc. In addition, even highly viscous sputum sample, which is usually difficult to be processed, could be directly processed without being homogenized. The time needed for pretreatment process could be shortened as low as approximately 15 minutes. The time needed for the conventional products widely used at clinical sites to perform amplification and detection is 30–60 minutes while the new amplification detection system requires approximately 15 minutes for swab and blood samples. Therefore, a total of approximately 30 minutes was achieved for the fully-automated testing. Also for sputum samples, the amplification and detection took only 20 minutes or less owing to the direct processing without homogenizing the sputum. The test chip taking advantage of the characteristics of LAMP has high sensitivity and high specificity and can realize simultaneous detection of up to 25 items including control. With the respiratory four-bacterium panel, the extraction performance of the present system is almost the same as that of conventional standard kits and sufficiently high detection sensitivity, specificity and cross-reactivity were confirmed. Use of this compact, rapid and simple system would realize simultaneous multi-item testing and random access testing near the patients. In the near future, we would like physicians and laboratory technicians to use our newly developed system as platform by expanding the lineup of multi-item test panels not only for infectious diseases but also for diagnosis of various diseases including cancers. And also we would like to contribute to medical field by assisting early detection and early treatment of a disease, treatment decisions and determination of dosage based on appropriate prognosis, and accelerating spread of nucleic acid-based diagnosis in Japan and the world with our system.

## Methods

### Overview of new fully automated testing system

**Figure 4:**
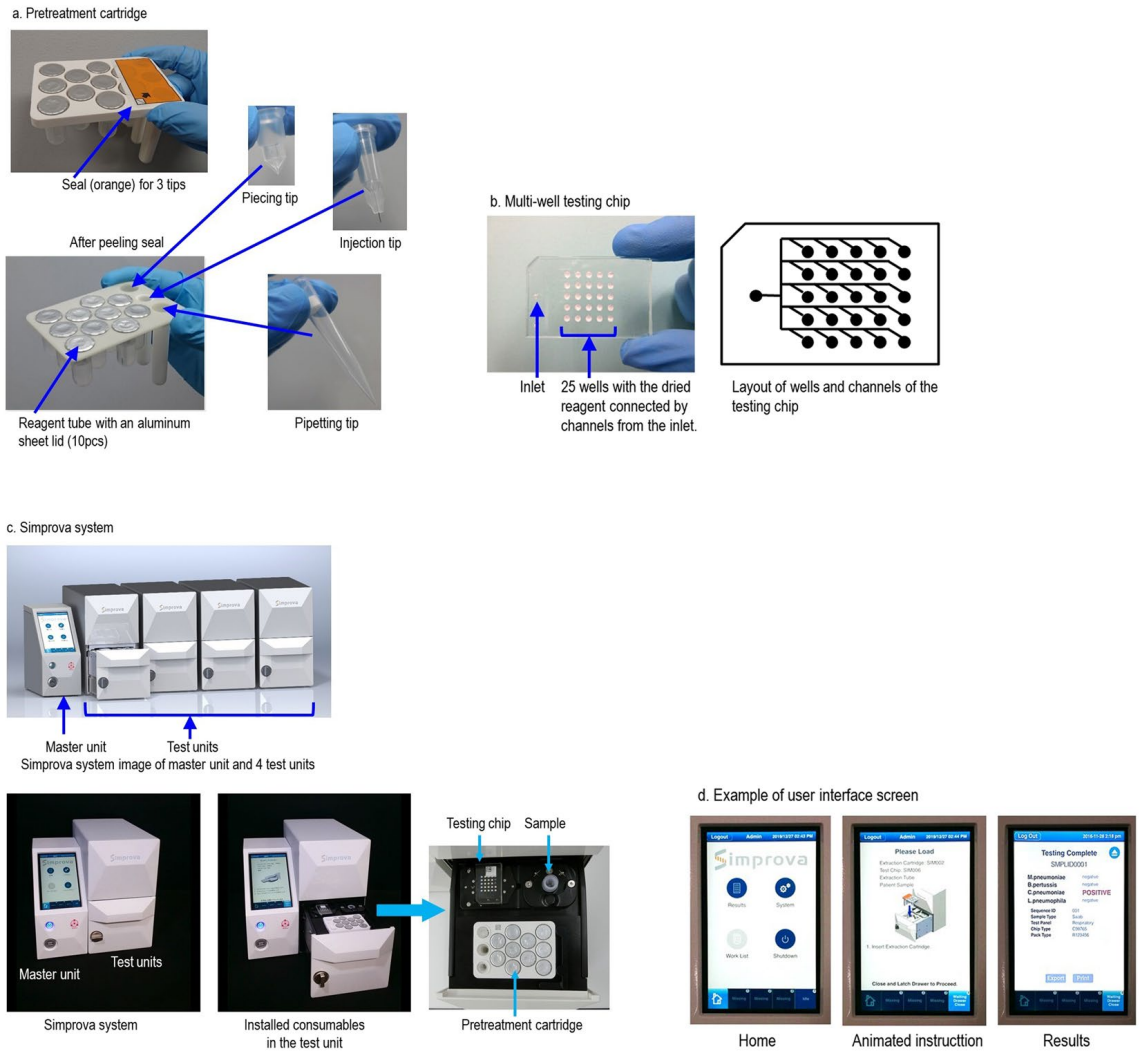
Overview of Simprova system. (**a**) pretreatment cartridge, (**b**) multi-well testing chip, (**c**) master unit and test unit. Up to four test units can be connected. (**d**) Example of user interface screen of the master unit.

The fully automated testing system that we developed consists of a master unit (controlling part) and test units (pretreatment and detection part) (Fig. 4c), and is used with 2 consumables, a pretreatment cartridge (Fig. 4a), a multi-well testing chip (Fig. 4b). The master unit, with its seven-inch touchscreen display, controls the test units. When the master unit is switched on, the test units are activated and mechanics are initialized. The user then inputs and confirms the test conditions on the display of the master unit, installs a sample, a pretreatment cartridge and a multi-well testing chip in the test unit according to animated instructions (Fig. 4d), and then touches a button to start the test. The entire processes from the extraction and purification of nucleic acid from samples to LAMP amplification, detection, analysis and result output are conducted in a fully automated way. As stated above, Simprova is a user-friendly system that needs no special training to operate.

The pretreatment cartridge contains consumables such as reagents to extract and purify nucleic acid from various samples, a pipette tip, a piercing tip to make a hole in the aluminum sheet lid on each reagent tube, and an injection tip. The multi-well testing chip has 25 reaction wells connected by channels. Each well contains dried reagent necessary for amplification and detection of nucleic acid by LAMP and up to 25 kinds of target genes including the control can be detected. This chip with dried reagent can be stored for more than 1 year at 2–30 °C. Turnaround time are expected to be approximately 30 minutes, which is the time that patients can wait at hospitals. In addition, the system is made compact enough to allow near-the-patient testing. Up to four test units can be connected to the master unit for random access test. Test results to use for assisting doctor’s diagnosis are automatically analyzed and shown on the master unit’s display or printed out. The system can also be connected to a Laboratory Information System (LIS) in hospital.

The testing system automatically conducts the following processes sequentially: (a) pretreatment to extract and purify nucleic acid from samples, (b) Injection of a solution containing the extracted and purified nucleic acid to wells in the multi-well testing chip, and (c) nucleic acid amplification by LAMP method and its detection in each well. The pretreatment and the amplification and detection process takes approximately 15 minutes, respectively. Therefore, the fully automated nucleic acid-based diagnosis would finish in a total of approximately 30 minutes. Each process is overviewed below.

#### Pretreatment process

**Figure 5:**
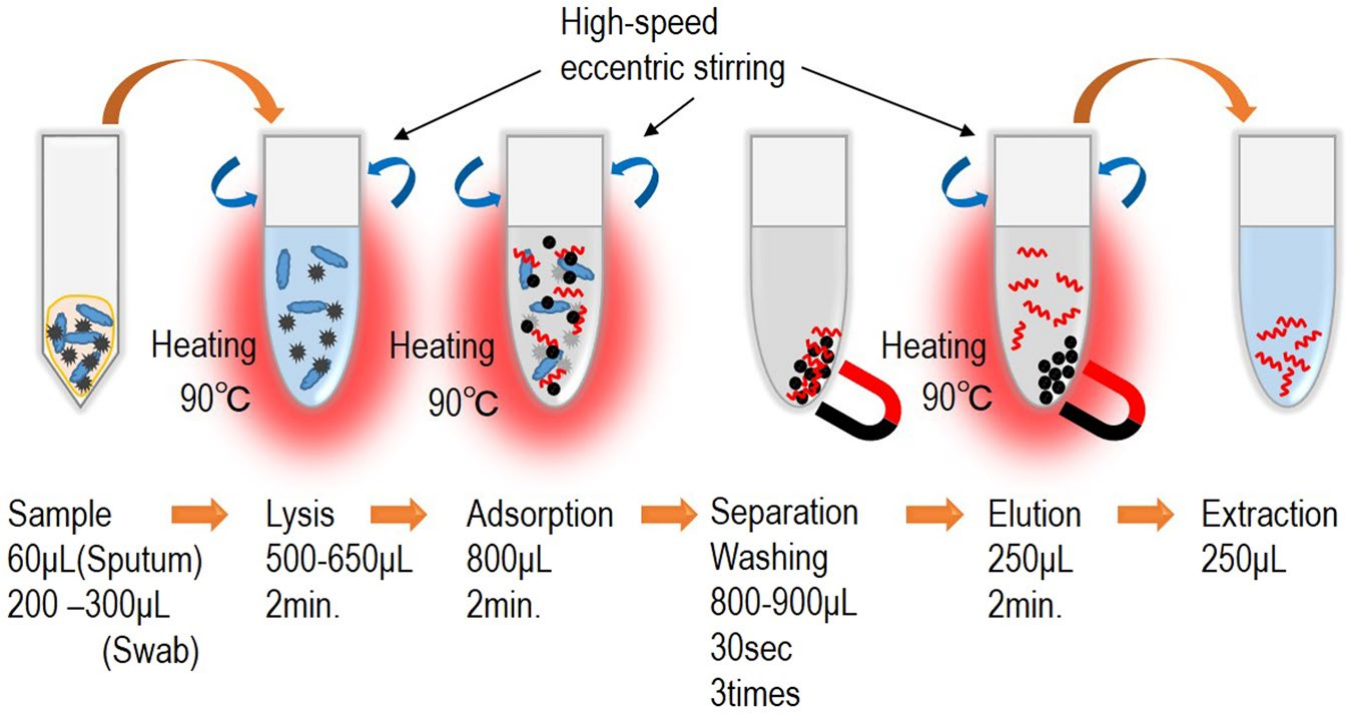
Outline of pretreatment process.

In the pretreatment process, a pretreatment cartridge containing a lysis buffer, binding buffer, wash buffer and elution buffer is used to extract and purify DNA or RNA from biological samples, which will be used as target for nucleic acid amplification. There are four base steps in the pretreatment process of the nucleic acid-based diagnosis: lysis, adsorption, washing and elution (Fig. 5). In the lysis step, the sample is lysed with chaotropic agent and surfactant and stirred at a high temperature to expose nucleic acid from biological samples into the solution. The system employs a high-speed eccentric stirring technique, which can reduce stirring time by 90% or more compared to conventional pipetting stirring. The exposed nucleic acid is adsorbed on the surface of magnetic silica beads through the high-speed eccentric stirring at the high temperature. Then, a neodymium magnet is used to separate the magnetic beads nucleic acid adsorbed from the solution containing the biological substance. After the separation, the solution other than the magnetic beads is discharged and the washing step begins. In the washing step repeated three times, alcohol concentration present in the washing solution is decreased to suppress influences to subsequent steps. The elution step is made highly efficient by increasing the temperature and using the high-speed eccentric stirring resulting in removing inhibition components against amplification. As stated above, we developed an original protocol where best reagents were used at each steps and a combination of heating and high-speed eccentric stirring was utilized to accelerate reactions. The protocol allowed us to obtain high-quality nucleic acid from various samples in approximately 15 minutes.

In these several steps of pretreatment, we have confirmed no possibility of the presence of aerosol from each reagent inside the test unit, which affects the testing process as a contaminant. Furthermore, the test unit has a structure that allows air to flow and evacuate aerosol toward a HEPA filter.

#### Process to inject nucleic acid solution to multi-well testing chip

The multi-well testing chip consists of 3 layers, a plastic substrate molded with polycarbonate that has 25 wells of approximately 5 μL volume each and channels measuring 50–100 μm wide and deep connecting the inlet and wells, a silicone-resin plate, and a plastic cover sheet made of polyethylene terephthalate. Each well has dried reagent for LAMP reaction. The nucleic acid solution extracted and purified in the pretreatment process is automatically injected to each well of the multi-well testing chip. The wells and channels of the chip are isolated from external atmosphere and depressurized to approximately 0.01 atm in the manufacturing process. The multi-well testing chip is packed in an aluminum vacuum bag to maintain the vacuum until it is used. An injection tip with a special needle is used to inject the nucleic acid solution into the wells of the testing chip. Once the needle of the injection tip is inserted into the inlet through the silicone resin layer as a valve, the nucleic acid solution in the injection tip is sucked in the wells via the channels for several tens of seconds by taking advantage of the pressure differential with the atmospheric air. This differential pressure injecting technique does not require a pump mechanism so that the unit can be made compact. Since the testing chip is a fully-closed system, the risk of contamination due to leakage of the sample or amplified nucleic acid is extremely low. The time of injecting the nucleic acid solution is as short as several tens of seconds, which contributes to the shortening of testing turnaround time.

#### LAMP amplification test process using multi-well testing chip

**Figure 6:**
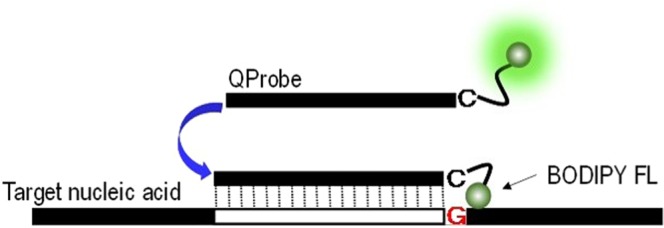
QProbe method. The fluorescence is quenched when the fluorescently labeled QProbe specifically binds to the target DNA amplified by LAMP. The fluorescent dye used is BODIPY FL, which has a maximum excitation wavelength of 503 nm and maximum emission wavelength of 513 nm.

**Figure 7:**
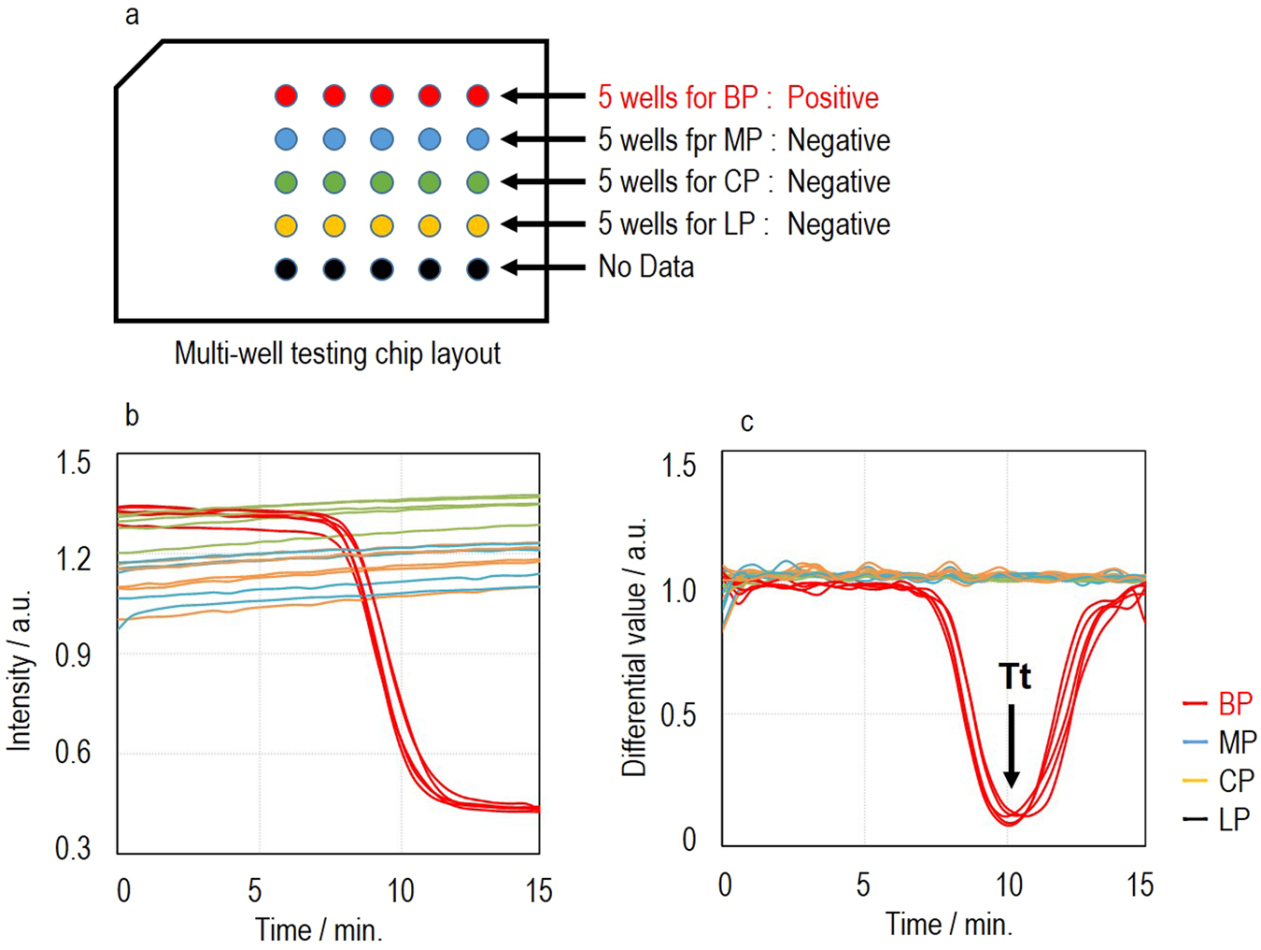
Signal image of LAMP. (**a**) Example of the multi-well testing chip layout to detect respiratory infectious diseases of BP, MP, CP and LP. (**b**) Example of detected signals. Fluorescence intensity decreases in amplified wells of BP (red). (**c**) Normalized differential curve of b. The minimum value can be obtained by differentiating the amplification curve. This minimum value is defined as the amplification time (Tt value).

The dried reagents for LAMP reaction in the wells are reconstituted with the nucleic acid solution injected and then the amplification of the nucleic acid begins by heating at a constant temperature. Each well has a common LAMP reagent, primer having a complementary sequence that specifically binds to a target gene and a probe necessary for the detection. The primers and probes with different sequences depending on the wells are used to detect different genes. For the detection, a quenching probe (QProbe) are applied (Fig. 6). The QProbe has cytosine at the end of the complementary sequence to the target gene. When it binds with the target nucleic acid, the fluorescence decreases due to the influence from guanine^15^. An optical detection unit consisting of an excitation light source, optical filters and detectors obtains the fluorescence intensity from the 25 wells of the testing chip in real time during amplification. Through the differential analysis of signals (Fig. 7b), an inflection point (minimum value) of the curve is obtained (Fig. 7c). The time at the minimum point is defined as amplification time (Tt) for example and used for automated determination where a positive result is output if Tt is within a preset cutoff value, which is set depending on the type of panel and sample. In the case of a respiratory bacteria panel for example, the cutoff value is 15 min for swab samples and 20 min for sputum samples, which is designed so that the test can be sensitive enough in clinical use.

Figure 7 shows the case where five wells are used to detect BP, MP, CP and LP respectively as a respiratory bacteria panel. The nucleic acid extracted in the pretreatment process is delivered into 25 wells. When the number of copies of target nucleic acid is sufficient to be delivered homogeneously to each well, amplification occurs in all five wells of BP as shown in Fig. 7. When it is quite low and close to the LoD, however, the number of copies delivered to each well may vary, and it is unlikely that all wells have a certain number of copies of target nucleic acid. When the target number of copies exceeding LoD exists only in one well, amplification occurs in such well. Therefore, the test result should be positive when one or more wells have positive results. The aim of using multiple wells is to increase the detection sensitivity according to the target. Furthermore, if one or more wells for different targets simultaneously give positive results, such test results likely indicate co-infection.

## Samples

**Figure 8:**
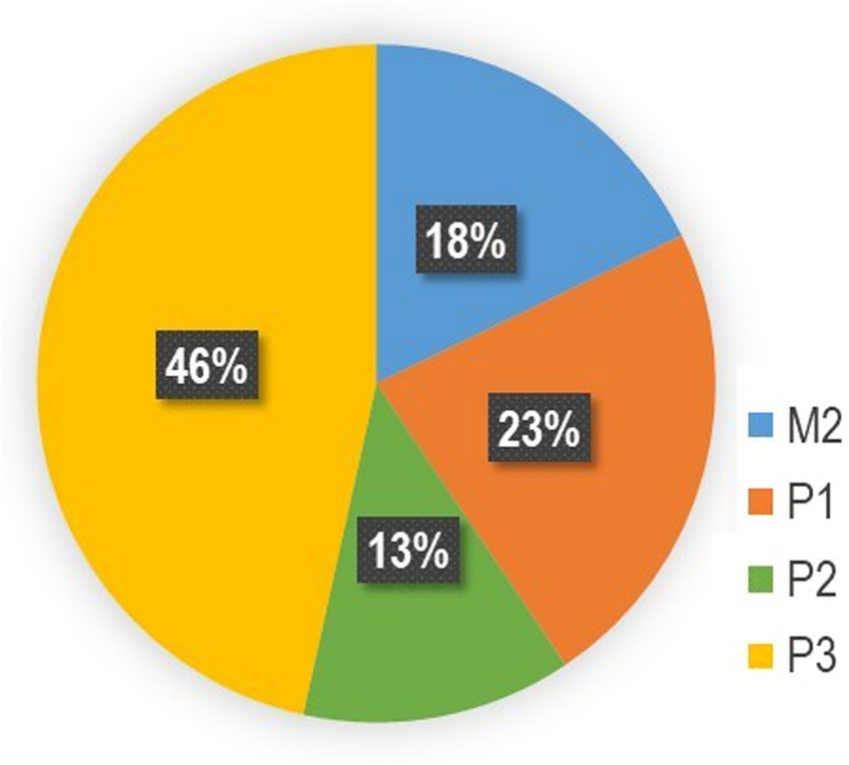
Miller and Jones’ classification^24^ of 101 sputum used in this study. Properties of sputum are M2; Small amount of purulent sputum included in viscous sputum, P1; Purulent portion comprising < one-third of all sputum, P2; Purulent portion comprising one-third–two-thirds of all sputum, P3; Purulent portion comprising > two-thirds of all sputum.

The performance of the new extraction protocol was evaluated by spiking bacterium, virus and yeast to a saline, throat swab, nasopharyngeal swab and whole blood. Swab samples of 200–300 μL each were provided by in-house volunteers following approval from the ethics committee, and whole blood was purchased from BizCom Japan (Tokyo, JAPAN). For direct extraction from sputum samples, 60 μL of sputum in various conditions was used (Fig. 8), and these were purchased from BizCom Japan (Tokyo, JAPAN). The bacterium, virus and yeast used in the spiking experiment are described in Supplementary Table 1. They were added to the samples so that 10^5^ to 10^7^ copies were produced when each pathogen and yeast were extracted. For a respiratory pathogens panel, 5 strains of *M. pneumoniae*, 10 strains of *B. pertussis*, 6 strains of *C. pneumoniae*, and 23 strains of *L. pneumophila* were used to check the specificity. For a cross-reactivity test, respiratory disease pathogens, pathogens of diseases other than respiratory diseases, and genome DNA extracted from human indigenous microbes of 1 ng described in Supplementary Table 2 were used.

### DNA extraction

We used QIAGEN DNA Mini Kit (QIAGEN) to extract nucleic acid from bacteria for comparison with the new extraction protocol. We also used QIAGEN RNeasy Mini kit (QIAGEN) to extract nucleic acid from virus. Extraction from each microbe was performed according to the manufacturer’s instructions.

### qPCR

The nucleic acid extraction performances of the new extraction method and the QIAGEN extraction method were evaluated with quantitative PCR. The quantitative PCR of each pathogen and yeast was performed by using an existing method (Supplementary Table 3)^16–23^. In the evaluation of the extraction performance, the same amount of bacteria or virus was used as input in both the new extraction method and the QIAGEN extraction method and the amount of the extracted target nucleic acid was measured with the quantitative PCR. The total amount of the extracted nucleic acid was calculated from the result of the PCR.

### LAMP

For detection of four kinds of bacteria that could cause respiratory infection diseases, a LAMP measurement system was developed for each bacterium (Supplementary Table 4). The DNA obtained by the new extraction method was injected to the multi-well testing chip by the above-mentioned differential pressure injecting technique. Reactions occurred for 15 min for swab samples and for 20 min for sputum samples under an isothermal condition (64 °C) and the LAMP amplification and detection unit in the new system measures fluorescence signal from each well by QProbe in real time to find fluorescence intensity decrease and hence amplification. Five wells were used for the detection of each item and the item was analyzed as “positive” if an amplification signal was detected from at least one of the five wells as mentioned above.

### Statement

All experiments and methods were performed in accordance with relevant guidelines and regulations. All experimental protocols were approved by the Eiken Chemical Research Ethics Committee (REC). Informed consent was obtained from all subjects and all methods were carried out in accordance with the relevant guidelines and regulations of REC.

## Supplementary information

Supplementary table S1

Supplementary table S2

Supplementary table S3

Supplementary table S4
