# Supplementary material for: Fully Automated Molecular Diagnostic System “Simprova” for Simultaneous Testing of Multiple Items
Source: Sci Rep. 2020 Mar 25;10:5409. doi: 10.1038/s41598-020-62109-5 (PMC7096461; doi:10.1038/s41598-020-62109-5)
Supplement: Supplementary file 1 — Supplementary table S1 [file 41598_2020_62109_MOESM1_ESM.pdf]

Title

Fully Automated Molecular Diagnostic System “Simprova” for Simultaneous Testing of Multiple Items

Author

Toshihiro Yonekawa , Hidetoshi Watanabe, Norimitsu Hosaka, Shohei Semba, Atsushi Shoji, Masaki Sato, Masato Hamasaki, Shota Yuki, Shiori Sano, Yuji Segawa*, Tsugunori Notomi

*Corresponding author; E-mail address, Yuji_Segawa@eiken.co.jp; Tel., +81-280-57-0717

Biochemical Research Laboratory II, Research & Development Division, Eiken Chemical Co., Ltd. 143 Nogi Nogimachi, Shimotsuga-gun, Tochigi, 329-0114, Japan

Supplementary Table 1. The bacterium, virus and yeast used in the spiking experiment.

**Bacteria**
Streptococcus pneumonia (ATCC49619), *M. pneumoniae* (ATCC15531), *B. pertussis* (clinical isolation), *C. pneumonia* (ATCC VR-2282), *L. pneumophila* (ATCC43703)
**Viruses**
Human Adenovirus 2 (ATCC VR-846), FluA H3N2 (clinical isolation)
**Yeast**
S. cerevisiae (BY611)(the National Bio-Resource Project (NBRP), Japan)
