# Supplementary material for: Fully Automated Molecular Diagnostic System “Simprova” for Simultaneous Testing of Multiple Items
Source: Sci Rep. 2020 Mar 25;10:5409. doi: 10.1038/s41598-020-62109-5 (PMC7096461; doi:10.1038/s41598-020-62109-5)
Supplement: Supplementary file 2 — Supplementary table S2 [file 41598_2020_62109_MOESM2_ESM.pdf]

Title

Fully Automated Molecular Diagnostic System “Simprova” for Simultaneous Testing of Multiple Items

Author

Toshihiro Yonekawa , Hidetoshi Watanabe, Norimitsu Hosaka, Shohei Semba, Atsushi Shoji, Masaki Sato, Masato Hamasaki, Shota Yuki, Shiori Sano, Yuji Segawa*, Tsugunori Notomi

*Corresponding author; E-mail address, Yuji_Segawa@eiken.co.jp; Tel., +81-280-57-0717

Biochemical Research Laboratory II, Research & Development Division, Eiken Chemical Co., Ltd. 143 Nogi Nogimachi, Shimotsuga-gun, Tochigi, 329-0114, Japan

Supplementary Table 2. No cross-reactivity with other pathogens

**Bacteria**
Acinetobacter baumannii, Acinetobacter lwoffii, Actinomyces israelii, Alcaligenes faecalis, Alcaligenes xylosoxidans, Bacteroides sp., Bordetella bronchiseptica, Bordetella parapertussis, Candida albicans, Chlamydophila pneumonia, Chryseobacterium indologenes, Corynebacterium pseudodiphtheriticum, Escherichia coli, Flavobacterium breve, Flavobacterium odoratum, Fusobacterium varium, Haemophilus influenza, Klebsiella pneumonia, Legionella pneumophila, Moraxella catarrhalis, Mycoplasma fermentans, Mycoplasma genitalium, Mycoplasma pneumonia, Mycoplasma salivarium, Neisseria meningitides, Peptococcus sp., Peptostreptococcus sp., Prevotella oralis, Propionibacterium avidum, Pseudomonas aeruginosa, Pseudomonas fluorescens, Pseudomonas stutzeri, Serratia marcescens, Staphylococcus aureus, Staphylococcus epidermidis, Streptococcus agalactiae, Streptococcus constellatus, Streptococcus equi, Streptococcus mitis, Streptococcus pneumonia, Streptococcus pyogenes, Veillonella parvula, Mycobacterium tuberculosis, Mycobacterium avium, Mycobacterium intracellulare, and Mycobacterium kansasii.
