# Supplementary material for: Fully Automated Molecular Diagnostic System “Simprova” for Simultaneous Testing of Multiple Items
Source: Sci Rep. 2020 Mar 25;10:5409. doi: 10.1038/s41598-020-62109-5 (PMC7096461; doi:10.1038/s41598-020-62109-5)
Supplement: Supplementary file 3 — Supplementary table S3 [file 41598_2020_62109_MOESM3_ESM.pdf]

Title

Fully Automated Molecular Diagnostic System “Simprova” for Simultaneous Testing of Multiple Items

Author

Toshihiro Yonekawa , Hidetoshi Watanabe, Norimitsu Hosaka, Shohei Semba, Atsushi Shoji, Masaki Sato, Masato Hamasaki, Shota Yuki, Shiori Sano, Yuji Segawa*, Tsugunori Notomi

*Corresponding author; E-mail address, Yuji_Segawa@eiken.co.jp; Tel., +81-280-57-0717

Biochemical Research Laboratory II, Research & Development Division, Eiken Chemical Co., Ltd. 143 Nogi Nogimachi, Shimotsuga-gun, Tochigi, 329-0114, Japan

Supplementary Table 3 Species and Target gene for qPCR

Species	Target gene	Reference
Streptococcus pneumoniae	LytA	17)
Human Adenovirus 2	hexon	18)
FluA H3N2	Matrix protein	19)
Saccharomyces cerevisiae	Mrp2	20)
Mycoplasma pneumoniae	16S rRNA	21)
Bordetella pertussis	IS*481*	22)
Chlamydophila pneumoniae	MOMP	23)
Legionella pneumophila	5S rRNA	24)
