# Supplementary material for: Fully Automated Molecular Diagnostic System “Simprova” for Simultaneous Testing of Multiple Items
Source: Sci Rep. 2020 Mar 25;10:5409. doi: 10.1038/s41598-020-62109-5 (PMC7096461; doi:10.1038/s41598-020-62109-5)
Supplement: Supplementary file 4 — Supplementary table S4 [file 41598_2020_62109_MOESM4_ESM.pdf]

Title

Fully Automated Molecular Diagnostic System “Simprova” for Simultaneous Testing of Multiple Items

Author

Toshihiro Yonekawa , Hidetoshi Watanabe, Norimitsu Hosaka, Shohei Semba, Atsushi Shoji, Masaki Sato, Masato Hamasaki, Shota Yuki, Shiori Sano, Yuji Segawa*, Tsugunori Notomi

*Corresponding author; E-mail address, Yuji_Segawa@eiken.co.jp; Tel., +81-280-57-0717

Biochemical Research Laboratory II, Research & Development Division, Eiken Chemical Co., Ltd. 143 Nogi Nogimachi, Shimotsuga-gun, Tochigi, 329-0114, Japan

Supplementary Table 4 Target genes, Primers and Probe for LAMP

Species	Target gene	Primers & Probe
M. pneumoniae	SDC	FIP: 5'-TCCCCCCTTTCATCCCACTCACTGGCTTGTTTACCCTGCTC-3'<br>BIP:5'-AAGTGCAAACGACTTACCCGGAAGGAGGCAATTTTGGCGGT-3'<br>F3:5'-TTGGTGGAAAACACGGCC-3'<br>B3:5'-TGTTGAGTGGGCTGGCATT-3'<br>LF:5'-GGTCACATACGCAAAGGTGTC-3'<br>LB:5'-CAAGTCCGACCAAAAGGC-3'<br>QP:5'-GGTCAAGTCCGACCAAAAGGCCACC（BODIPY-FL）-3'
B. pertussis	ptxA	FIP: 5'-CGACTTTGCGCCGAAGGAGACATCCCGCTACTGCAATCC-3'<br>BIP: 5'-GCGATGGTACCGGTCACCGCGTTTTGATGGTGCCTATTTTACG-3'<br>F3: 5'-AACCCTCGATTCTTCCGT-3'<br>B3: 5'-TCCCGTCTTCCCCTCTG-3'<br>LF: 5'-GGAGCGTTCATGCCGTG-3'<br>LB: 5'-CCCCTGCCATGGTGTGA-3'<br>QP: 5'-CGTCCGGACCGTGCTGACCC（BODIPY-FL）-3'
C. pneumoniae	53kDa protein	FIP: 5'-GCCTGTTGCCAAAATTGAGTGAACATAGGCTCAATTTCAGAAAGAAGTCGG-3'<br>BIP:5'-GCCTCAAAACAAACAGGCGAGTCTA-GGATTTGAGCGCCCAGC-3'<br>F3:5'-CTCTCGGAGATGCAACAAAAC-3'<br>B3:5'-GCTGATTGCGGCATACGC-3'<br>LF:5'-TCATATCAGCCGCAGCCTG-3'<br>LB:5'-CTAATGAAATGACTCAAAAAGCTACC-3'<br>QP:5'-TCAGCCGCAGCCTGCAGTTTTCC（BODIPY-FL）-3'
L. pneumophila	16S rRNA	FIP: 5'-CCCTCAGGCCTTCTTCACACACCTACGGGAGGCAGCAGTG-3'<br>BIP:5'-CAGTGGGGAGGAGGGTTGATAGGTGGAGTTAGCCGGTGCTTC-3'<br>F3:5'-TGAGAGGATGACCAGCCA-3'<br>B3:5'-GCTCGCACCCTCCGTATT-3'<br>LF:5'-GGTTGCCCCCATTGTCC-3'<br>LB:5'-TTAACTGGACGTTACCCACAGA-3'<br>QP:5'-TGGATCAGGGTTGCCCCATTGTCCAATATTCCC（BODIPY-FL）-3'
